# Maintenance of drug metabolism and transport functions in human precision-cut liver slices during prolonged incubation for 5 days

**DOI:** 10.1007/s00204-016-1865-x

**Published:** 2016-10-07

**Authors:** Viktoriia Starokozhko, Suresh Vatakuti, Bauke Schievink, Marjolijn T. Merema, Annika Asplund, Jane Synnergren, Anders Aspegren, Geny M. M. Groothuis

**Affiliations:** 1 Division of Pharmacokinetics Toxicology and Targeting, Groningen Research Institute for Pharmacy, University of Groningen, Groningen, The Netherlands; 2 Department of Clinical Pharmacy and Pharmacology, University of Groningen, University Medical Center Groningen, Groningen, The Netherlands; 3 School of Biosciences, Systems Biology Research Center, University of Skövde, 54128 Skövde, Sweden; 4 Takara Bio Europe AB, Gothenburg, Sweden

**Keywords:** Human precision-cut liver slices, Metabolism, Drug transport, Transcriptomics, Prolonged incubation

## Abstract

Human precision-cut liver slices (hPCLS) are a valuable ex vivo model that can be used in acute toxicity studies. However, a rapid decline in metabolic enzyme activity limits their use in studies that require a prolonged xenobiotic exposure. The aim of the study was to extend the viability and function of hPCLS to 5 days of incubation. hPCLS were incubated in two media developed for long-term culture of hepatocytes, RegeneMed^®^, and Cellartis^®^, and in the standard medium WME. Maintenance of phase I and II metabolism was studied both on gene expression as well as functional level using a mixture of CYP isoform-specific substrates. Albumin synthesis, morphological integrity, and glycogen storage was assessed, and gene expression was studied by transcriptomic analysis using microarrays with a focus on genes involved in drug metabolism, transport and toxicity. The data show that hPCLS retain their viability and functionality during 5 days of incubation in Cellartis^®^ medium. Albumin synthesis as well as the activity and gene expression of phase I and II metabolic enzymes did not decline during 120-h incubation in Cellartis^®^ medium, with CYP2C9 activity as the only exception. Glycogen storage and morphological integrity were maintained. Moreover, gene expression changes in hPCLS during incubation were limited and mostly related to cytoskeleton remodeling, fibrosis, and moderate oxidative stress. The expression of genes involved in drug transport, which is an important factor in determining the intracellular xenobiotic exposure, was also unchanged. Therefore, we conclude that hPCLS cultured in Cellartis^®^ medium are a valuable human ex vivo model for toxicological and pharmacological studies that require prolonged xenobiotic exposure.

**Electronic supplementary material:**

The online version of this article (doi:10.1007/s00204-016-1865-x) contains supplementary material, which is available to authorized users.

## Introduction

In the past decades, development of new toxicity models that reduce or replace animal use gained much scientific interest. These methods include 2D and 3D cultures of freshly isolated cells, differentiated stem cells, or cell lines, either in monoculture or in co-cultures. Currently, it is generally assumed that 3D co-culture models reflect organ functions more closely than 2D monocultures. Precision-cut liver slices (PCLS) have already shown to be a functional and efficient liver model in various pharmacological and toxicological studies (de Graaf et al. 2007, 2010; Elferink et al. 2008; Vickers and Fisher 2013). For example, PCLS have been widely used to study metabolic pathways of xenobiotics, to obtain kinetic data on metabolism and transport, or to study drug–drug interactions related to inhibition or induction of various metabolic enzymes (de Graaf et al. 2006; Lake and Price 2013; Olinga et al. 2008; Pfeiffer and Metzler 2004). In addition, many 3D liver models have been developed, including hepatocytes mono-cultures and co-culture systems with hepatocytes and non-parenchymal liver cells (Bell et al. 2016; Godoy et al. 2013). The main advantage of the PCLS model above the other 3D liver models is the presence of all liver cells types in their natural relative ratio and tissue-matrix configuration, allowing cell–cell and cell–matrix interactions, with all vital liver functions represented (de Graaf et al. 2007; Soldatow et al. 2013). Moreover, the use of human PCLS (hPCLS) enables a direct in vitro identification of pharmacological and toxicological mechanisms relevant for human exposure (Vickers and Fisher 2013).

The toxicity of a xenobiotic compound often directly depends on its biotransformation, which leads to detoxification or toxification of the parent compound. Therefore, presence and maintenance of the activity of the metabolic enzymes as well as transporter proteins, that transport the parent compound as well as metabolites in and out of the cells, is a key requirement for an in vitro liver model from a toxicological point of view (Lerche-Langrand and Toutain 2000). Even though fresh PCLS contain the whole range of phase I and phase II metabolic enzymes and their viability can be maintained for several days (Vickers et al. 2004, 2011), the decline in xenobiotic metabolizing enzyme activities in culture, although not as rapid as in isolated hepatocytes in conventional 2D cultures, is still a major restriction (de Graaf et al. 2010; Ioannides 2013; Lake and Price 2013; Lerche-Langrand and Toutain 2000; Vickers et al. 2011). Although this decline does not prevent the use of PCLS in cytochrome P450 induction studies or acute toxicity studies, their use in (sub)chronic toxicology studies, however, may yield data that are not representative of the in vivo situation (Ioannides 2013; Lake and Price 2013). Thus, optimization of PCLS metabolic functions in culture is an important factor for toxicological studies that require a prolonged drug exposure (Lake and Price 2013; Olinga and Schuppan 2013).

Improved viability and functionality of the slices can be achieved by improving culture conditions such as medium composition (Olinga et al. 1997; Starokozhko et al. 2015). For example, a recent study on rat PCLS showed that the medium composition has a large impact on tissue viability and functions following 5 days of incubation (Starokozhko et al. 2015). It is generally known that for a proper prediction of drug disposition and toxicity, it is very important to use human cells or tissues because of large species differences in these functions (Hadi et al. 2013). However, full maintenance of these functions for more than 1–2 days has not yet been achieved in hPCLS (Renwick et al. 2000; VandenBranden et al. 1998; Vickers et al. 2011). Therefore, the aim of this study was to extend the functional viability of hPCLS to 5 days of incubation by investigating the stability of metabolic enzyme activities, synthesis functions, as well as the expression of the genes responsible for xenobiotic metabolism and transport in hPCLS during prolonged incubation in three different culture media. Williams’ Medium E (WME) was chosen as a standard cell culture medium that is commonly used for PCLS incubation (Duryee et al. 2014; Jetten et al. 2014; Westra et al. 2014). As a second medium we chose RegeneMed^®^, which was designed and used for long-term culture of primary human liver cells (Kostadinova et al. 2013) and which we tested on rat PCLS before (Starokozhko et al. 2015). As a third medium, we tested Cellartis^®^ Hepatocyte Maintenance Medium (Takara Bio Europe AB), which was originally designed as maintenance medium for induced pluripotent stem-cell-derived hepatocytes, to maintain viability, differentiation, and liver functions. We characterized the viability and morphological and functional changes (albumin synthesis) in hPCLS during 5 days of incubation. Maintenance of phase I and II metabolism was studied both on gene expression and functional levels. Moreover, we performed transcriptomic analysis of the gene expression using microarrays and focused on the expression of genes involved in drug metabolism, transport and toxicity, oxidative stress, and fibrogenesis.

## Materials and methods

### Human livers

Human liver material was obtained from the healthy parts of liver tissue of five individual patients, undergoing hepatectomy for the removal of carcinoma, from donor liver tissue after reduced size liver transplantation or from liver tissue donated after cardiac death but not suitable for transplantation (See Table 4 for details, Supplementary materials). The experimental protocols were approved by the Medical Ethical Committee of the University Medical Center Groningen.

### Preparation and incubation of human PCLS

hPCLS were prepared as described previously by de Graaf et al. with minor modifications (de Graaf et al. 2010). hPCLS of 5 mm in diameter and approximately 5 mg wet weight was used in this study. Slices were pre-incubated for 1 h at 37 °C in a 12-well plate filled with 1.3 ml of WME (Gibco, Life Technology) saturated with 80 % O2/5 % CO2 while gently shaking 90 times/min. Thereafter, they were transferred to another 12-well plate filled with 1.3 ml of three different media saturated with 80 %O2/5CO2: WME (with l-glutamine, Invitrogen, Paisly, Scotland) supplemented with 25 mM glucose and 50 mg/ml gentamycin (Invitrogen), RegeneMed^®^ medium: WME supplemented with RegeneMed^®^ additives (L3STA), antibiotics (L3MAB) and supplements (L3STS) in ratio 100:15.1:1:2.5 (RegeneMed^®^, San Diego, CA, USA) or Cellartis^®^ Hepatocyte Maintenance Medium: WME supplemented with Cellartis^®^ Hepatocyte Maintenance Medium Supplements (Cat. No. Y30051, Takara Bio Europe AB, Gothenburg, Sweden) and 50 mg/ml gentamycin. PCLS were incubated for 5 days with medium being refreshed daily.

### ATP and protein content of hPCLS

Viability of hPCLS was determined at different time points (0, 24, 48, 72, 96, and 120 h) by means of the ATP content of the hPCLS as described previously using the ATP Bioluminescence Assay Kit CLS II (Roche, Mannheim, Germany) (de Graaf et al. 2010). Protein content of the hPCLS was measured according to Lowry by using the Bio-Rad DC Protein Assay (Bio-Rad, Munich, Germany) using a bovine serum albumin calibration curve (Lowry et al. 1951) as previously described (Starokozhko et al. 2015).

### Paraffin sections of hPCLS

hPCLS were collected after each experimental time point and fixed in 4 % formaldehyde in phosphate buffered saline (PBS) solution for 24 h at 4 °C and stored until analysis in 70 % ethanol at 4 °C. After dehydration in alcohol and xylene, the slices were embedded in paraffin and sectioned (4-µm-thick sections) perpendicular to the surface of the slice.

### Morphological assessment

Morphological assessment of hPCLS was performed on paraffin sections, stained with hematoxylin and eosin (Klinipath, the Netherlands) (H&E) as described previously (de Graaf et al. 2000).

### Periodic acid-schiff staining (PAS) and Sirius red staining

The glycogen content of hPCLS was determined by the periodic acid-Schiff (PAS) staining as described previously by Schaart et al. (2004), with some modifications as described before (Starokozhko et al. 2015). Staining for fibrillary collagen was performed on 4-µm paraffin sections using picrosirius red (Sigma, Gillingham, UK). In brief, slices were deparaffinized and stained in picrosirius red dye (0.1 % picric acid). Thereafter, sections were washed two times in acidified water (5 ml/L glacial acid), dehydrated, and embedded in Depex.

### Functional characterization of hPCLS

#### Phase I and II metabolism

To test the activities of different CYP isoenzymes, hPCLS were incubated for 3 h with a drug cocktail containing 10 µM phenacetin (CYP1A), 10 µM bupropion (CYP2B6), 50 µM mephenytoin (CYP2C19), 10 µM diclofenac (CYP2C9), 10 µM bufuralol (CYP2D6), and 5 µM midazolam (CYP3A) in medium without phenol red. Medium was collected and stored at −80 °C until further analysis. Metabolite concentrations were measured by Pharmacelsus (Germany) by LC/MS according to in house protocols. The metabolite production was normalized per mg protein and per hour.

To assess both phase I and II metabolism, hPCLS were incubated with 100 µM 7-ethoxycoumarin (7-EC) for 3 h. 7-EC is metabolized first to 7-hydroxycoumarin (7-HC) by Cytochrome P450, which further undergoes glucuronidation [7-hydroxycoumarin-glucuronide (7-HC-G)] and sulfation (7-hydroxycoumarin-sulfate (7-HC-S). Furthermore, to measure directly phase II metabolism activity, hPCLS were exposed directly to 100 µM of 7-HC (Sigma-Aldrich, St. Louis, MO, USA) for 3 h. Medium was collected and stored at −20 °C until further analysis by HPLC as described before (de Kanter et al. 2004), using 7-EC, 7-HC, 7-HC-G, and 7-HC-S as standards. The metabolite production was normalized per mg protein and per hour.

### Albumin production

Albumin production was measured using the Human Albumin ELISA kit (Bethyl Laboratories, Mongomery, USA) according to the supplier’s protocol. In brief, medium was collected every day and stored at −20 °C until analysis. Samples were diluted if necessary. The amount of albumin was calculated based on a standard curve of human albumin generated as a four-parameter curve fit. Values are expressed as ng albumin produced per hour, per mg total protein.

### RNA isolation

RNA was isolated from slices incubated for 120 and the 0 h (control samples). RNA isolation was performed using the Maxwell^®^ 16 LEV Total RNA purification kit (Promega, the Netherlands) with Maxwell^®^ 16 LEV Instrument. Immediately after isolation, the RNA quality was assessed by measuring the 260/230 and 260/280 ratios, and the concentration was measured with the ND-1000 spectrophotometer (Fisher Scientific, Landsmeer, the Netherlands). The quality (RIN value) and quantity of the RNA were further determined by high-throughput Caliper GX LabChip RNA kit (Caliper) before the RNA amplification.

### Amplification, labeling and hybridization of RNA samples

Ambion Illumina Total Prep RNA kit was used to transcribe 300 ng RNA to cRNA according to the manufacturer’s instructions. A total of 750 ng of cRNA was hybridized at 58 °C for 16 h to the Illumina HumanHT-12 v4 Expression BeadChips (Illumina, San Diego, CA, USA). BeadChips were scanned using Iscan software, and raw IDAT files were generated.

### Preprocessing of gene expression data

GenomeStudio software (Illumina) was used to generate raw expression values from the IDAT files. The ArrayAnalysis Web service was used for further preprocessing the data, which uses the package “lumi,” for the R software environment (R Foundation for Statistical Computing, Vienna, Austria; Eijssen et al. 2013). Raw gene expression data were background-corrected (bgAdjust), variance-stabilized (VST), and normalized by quantile normalization. Differentially expressed genes in slices incubated for 120 h with Cellartis^®^ medium versus the control slices (0 h) were identified using the moderated *t* test in the ‘limma’ package of the R software environment (Ritchie et al. 2015). Genes that are regulated with a criterion of fold change of 1.5 (≤ or ≥1.5), and FDR-corrected *p* value ≤0.05 (Benjamini and Hochberg method) was chosen for pathway analysis.

### Gene expression pattern analysis

Gene expression pattern analysis of the data was performed by GEDI software (default settings) and metagene (set of genes whose expression change similarly in the incubated samples compared to control samples) signature of each sample is represented in a grid of 26 × 25 tiles; each of the tiles contains genes that are highly correlated with each other (Eichler et al. 2003). The tiles are arranged such that each tile is also correlated with the adjacent tiles. Thus, it allows a global first-level analysis of the transcriptomic changes due to incubation.

### Pathway analysis

Pathway analysis (canonical metabolic and signaling pathways) was performed to identify the significantly regulated pathways using QIAGEN’s Ingenuity^®^ Pathway Analysis (IPA^®^, QIAGEN Redwood City, CA, USA). The annotations of the genes related to metabolism, transport, and toxicity processes such as fibrosis and stress response genes were retrieved from the Ingenuity knowledgebase.

### Statistics

Three to four different human livers were used for each experiment, using slices in triplicates from each liver. Statistical testing was performed with two way repeated measures ANOVA with the individual human as random effect. We performed a Tukey HSD post hoc test for pairwise comparisons. A *p* value of ≤0.05 was considered to be significant. In all graphs the mean values and standard error of the mean (SEM) are shown. All statistical analysis was performed using R version 3.2.2 (R Foundation for Statistical Computing, Vienna, Austria).

## Results

### Viability

**Fig. 1:**
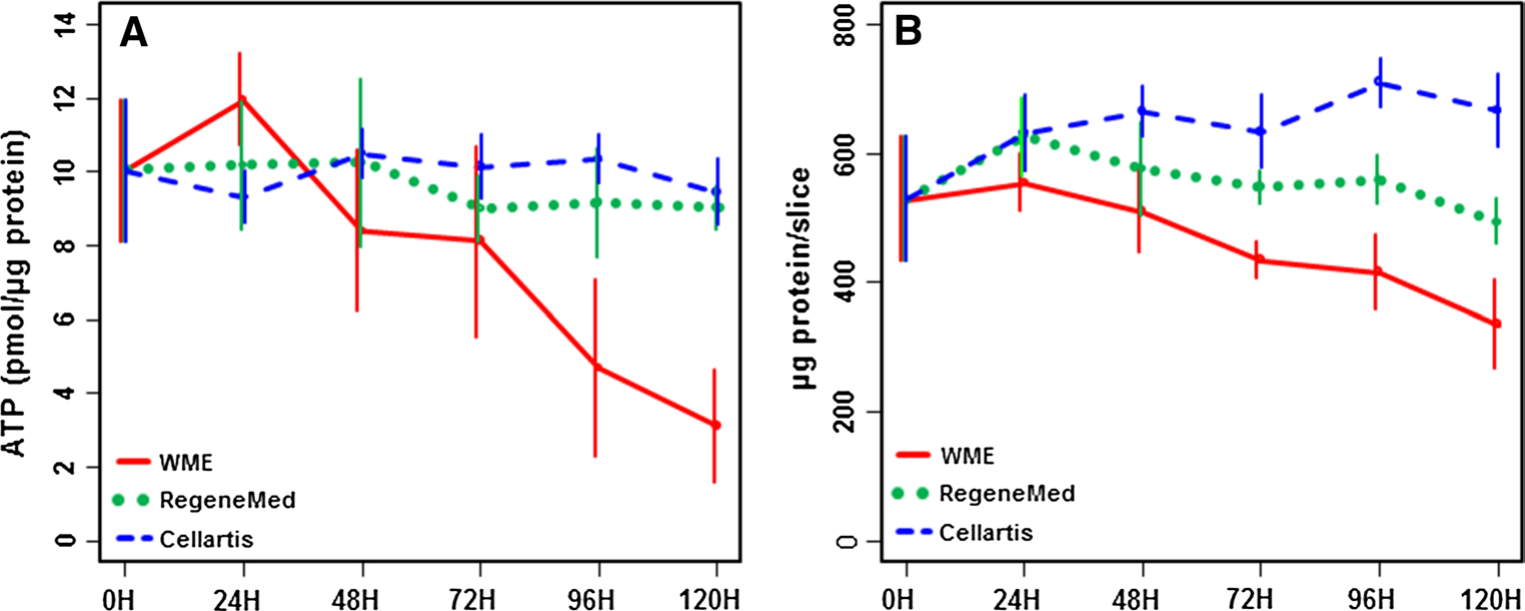
ATP (**a**) and protein (**b**) content in hPCLS during 120 h incubation in three different media [WME (*red line*), RegeneMed^®^ (*green dotted line*), and Cellartis^®^ (*blue dashed line*)]. Data represent the average ± SEM of four experiments (four different livers), using three hPCLS for each group in every experiment (color figure online)

The viability of the hPCLS during incubation for 120 h was assessed by ATP content (Fig. 1a). hPCLS incubated in RegeneMed^®^ and Cellartis^®^ medium maintained the ATP level at least up to 120 h of incubation. However, ATP content in hPCLS incubated in WME decreased significantly over time (*p* = 0.03). The protein content remained constant in slices incubated in RegeneMed^®^ during 5 days of incubation, whereas it increased somewhat in slices incubated in Cellartis^®^ (*p* = 0.04) and significantly decreased in slices incubated in WME (*p* = 0.005) (Fig. 1b).

### Morphological examination of hPCLS

**Fig. 2:**
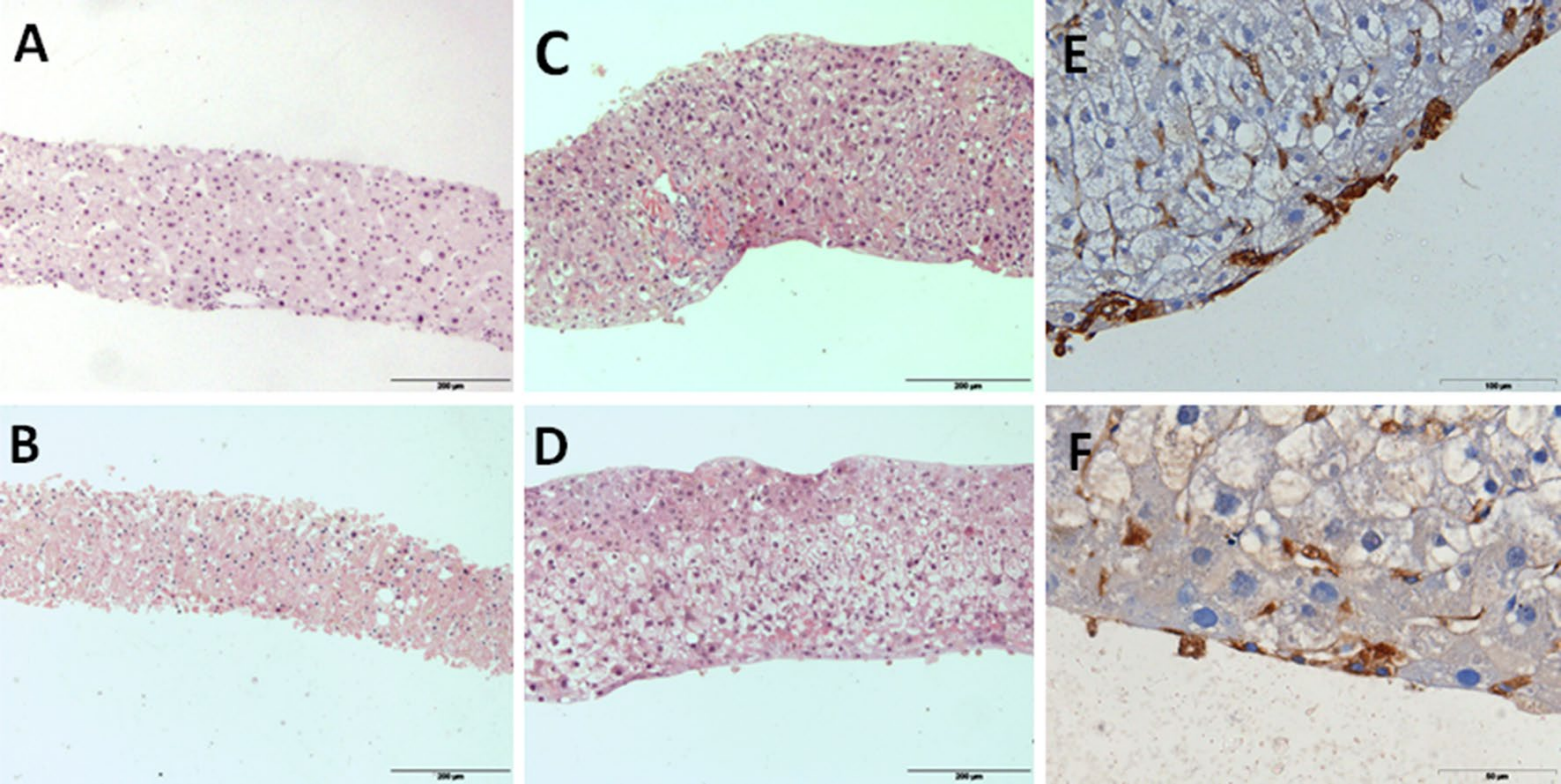
Hematoxylin–eosin staining of cross sections of hPCLS at 0 h (**a**) and incubated 120 h in WME (**b**), RegeneMed^®^ (**c**) or Cellartis^®^ (**d**). Vimentin staining of the new cell layer around the slice incubated for 120 h in RegeneMed^®^ (**e**) or Cellartis^®^ (**f**). Representative images are shown. *Scale bar* 200 µm for **a**–**d**, 100 µm for **e** and 50 µm for **f**

The viability of hPCLS following incubation up to 120 h was also assessed by histomorphology (Fig. 2). After the slicing procedure, hPCLS had normal tissue architecture with all liver cell types present. Following prolonged incubation in WME, substantial necrotic zones with pyknotic nuclei were observed in the slices. On the contrary, hPCLS incubated in RegeneMed^®^ or Cellartis^®^, contained viable hepatocytes with occasional small necrotic areas. Slices showed a higher cell density due to substantially narrowed sinusoids after 120 h of incubation. In the hPCLS incubated in Cellartis^®^ medium, hepatocytes contained unstained areas, probably due to glycogen deposits (see below). These were also visible in the slices incubated in RegeneMed^®^, although less pronounced. The thickness of the slices incubated in RegeneMed^®^ or Cellartis^®^ increased during incubation (Fig. 2). Moreover, the formation of a new cell layer was observed during prolonged incubation of hPCLS in RegeneMed^®^ and Cellartis^®^, which was positive for vimentin (Fig. 2e and f).

**Fig. 3:**
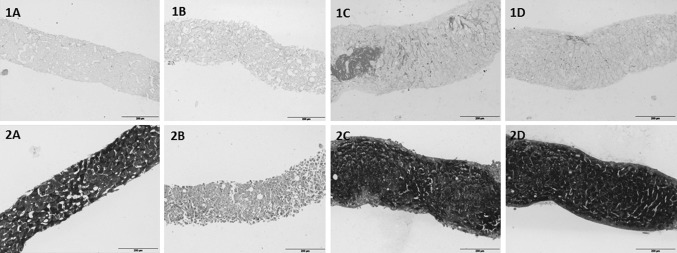
Cross sections of hPCLS at 0 h (**a**) and incubated 120 h in WME (**b**), RegeneMed^®^ (**c**) or Cellartis^®^ (**d**) stained with Sirius Red (1) or PAS (2). Representative images are shown. *Scale bar* 200 µm

Sirius red staining revealed an increased collagen deposition in slices incubated in all three media. In non-incubated slices, collagen was deposited mainly around the portal vein, bile ducts, and hepatic vein, and only a few very thin collagen fibers were observed in some areas of the parenchyma. In slices incubated in Cellartis^®^ medium, collagen fibers in the parenchyma became thicker and more visible. Moreover, occasional nodes of collagen were observed, which were mostly located in the portal area (Fig. 3, 1D). These changes were substantially more pronounced in slices incubated in RegeneMed^®^, where large nodes of collagen located in the portal areas, as well as in the parenchyma were observed (Fig. 3, 1C). Slices incubated in WME also showed an increase in collagen deposition in the parenchyma (Fig. 3, 1B).

Slices fixed at 0 h showed high and homogeneous glycogen deposition. Following 5 days of incubation in RegeneMed^®^ and Cellartis^®^, but not in WME, hPCLS maintained the ability to synthesize and deposit glycogen, which indicates an adequate oxygen as well as nutrient supply and good energy balance during incubation. An intensive glycogen deposition in the areas where large vacuoles in hepatocytes were seen indicates that those vacuoles are filled with glycogen. hPCLS incubated in WME did not contain glycogen after 5 days of incubation (Fig. 3, 2A–2D).

### Phase I and phase II metabolism

**Fig. 4:**
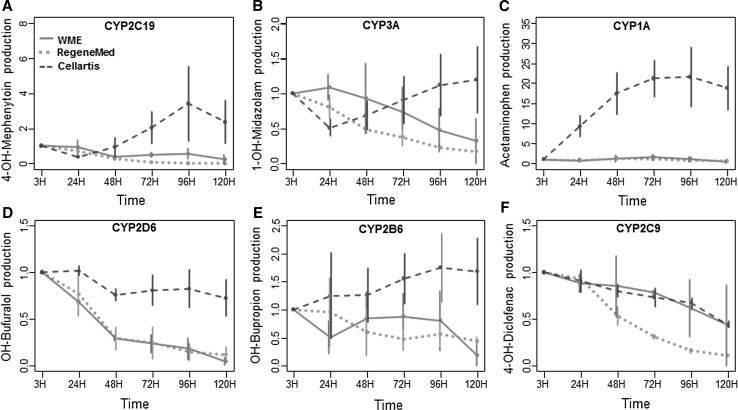
Phase I metabolite production of mephenytoin (**a**), midazolam (**b**), phenacetin (**c**), bufuralol (**d**), bupropion (**e**), and diclofenac (**f**) during 5 days by hPCLS incubated in WME (*red line*), RegeneMed^®^ (*green dotted line*), and Cellartis^®^ (*blue dashed line*). Date are expressed as relative values ± SEM to the value at 0–3 h incubation. 3 (WME and Cellartis^®^) or 2 (RegeneMed^®^) donor livers were used for each study, using three hPCLS for each group in every experiment (color figure online)

The activities of metabolic enzymes in hPCLS from different donors showed large inter-individual variation as expected based on well-described variations in the human population due to disease conditions, exposure to other drugs and food components and polymorphisms in drug metabolizing enzymes. Therefore, metabolite production levels at different days during incubation are expressed as relative to the value of the fresh hPCLS of the corresponding liver (Fig. 4).

The incubation time had different effects on metabolic enzymes in hPCLS incubated in the different media. Overall, the three media differed significantly in their effect on the activity of most of the tested CYP isoforms (CYP2C19: *p* < 0.01, CYP1A: *p* < 0.001, CYP2D6: *p* < 0.001, CYP2B6: *p* < 0.01). In WME the activity of CYP2D6, CYP2B6, and CYP3A at 120 h in hPCLS was lower compared to 3 h value, whereas the activities of CYP2C9, CYP2C19, and CYP1A remained constant. In RegeneMed^®^ the activity of four of the CYP isoforms declined in time (*p* < 0.01 for CYP2D6 and CYP3A, *p* < 0.001 for CYP2C9 and CYP2C19). The activity of CYP2B6 and CYP1A, however, were not significantly changed. On the contrary, in Cellartis^®^ medium the activity of all tested cytochrome P450 isoforms did not decline during 120 h in hPCLS incubated, with a slight decrease of CYP2C9 as the only exception. Interestingly, the activity of CYP1A strongly increased over time in slices incubated in Cellartis^®^ medium (*p* < 0.01 for overall effect of time).

**Fig. 5:**
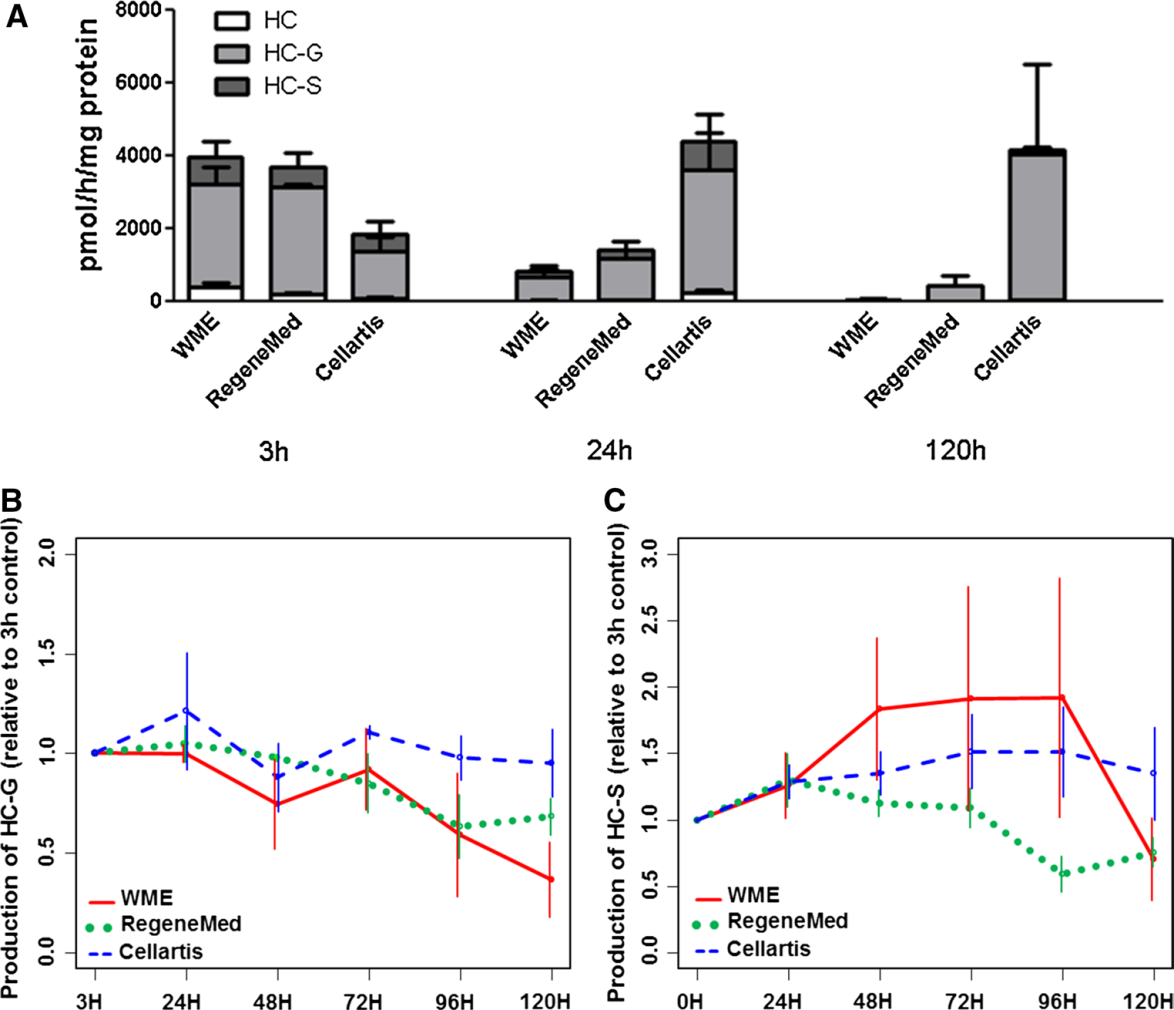
Phase I and II metabolism of 7-EC (**a**) and Phase II metabolism of 7-HC (**b**, **c**) during 5 days by hPCLS incubated in WME (*red line*), RegeneMed^®^ (*green dotted line*), and Cellartis^®^ (*blue dashed line*). Data are expressed as relative values (±SEM) to the value at 0–3 h incubation (color figure online)

7-EC is metabolized in the human liver mainly by CYP1A2 and CYP2E1 to 7-HC, which undergoes further glucuronidation (7-HC-G) and sulfation (7-HC-S) by uridine UDP-glucuronyltransferases (UGTs) and sulfotransferases (SULTs), respectively. The total phase I metabolic rate of 7-EC is calculated as the total amount of 7-HC, 7-HC-G, and 7-HC-S produced. The results show that the medium composition has a significant effect on 7-EC metabolism by hPCLS (*p* < 0.001), in line with the findings with the drug cocktail. Thus, metabolite production of 7-EC decreased substantially already after 24 h in slices incubated in WME or RegeneMed^®^, with a further decline over 120 h of incubation. hPCLS incubated in Cellartis^®^ medium, on the other hand, had a constant or even increasing overall metabolite production over time (Fig. 5a).

To study phase II metabolism separately, the slices were exposed to 7-HC. Phase II metabolism of 7-HC was shown to be affected by both time and medium composition (Fig. 5b, c). For example, production of both 7-HC-G and 7-HC-S from 7-HC slightly declined over time in hPCLS incubated in RegeneMed^®^, whereas their production remained constant in Cellartis^®^ medium. The SULT activity was stable also in hPCLS incubated in WME, but the UGT activity, however, declined over time. The phase II metabolism of 7-HC in RegeneMed^®^ and WME formed after oxidation of 7-EC decreased apparently due to a decrease in Phase I metabolism. Moreover, it showed a different pattern in Cellartis^®^ medium toward higher production of glucuronides and lower sulfation rates (Fig. 5a) compared to the metabolism of 7-HC added directly to hPCLS, where sulfation rates in Cellartis^®^ medium remained stable over time (Fig. 5c).

### Albumin synthesis

**Fig. 6:**
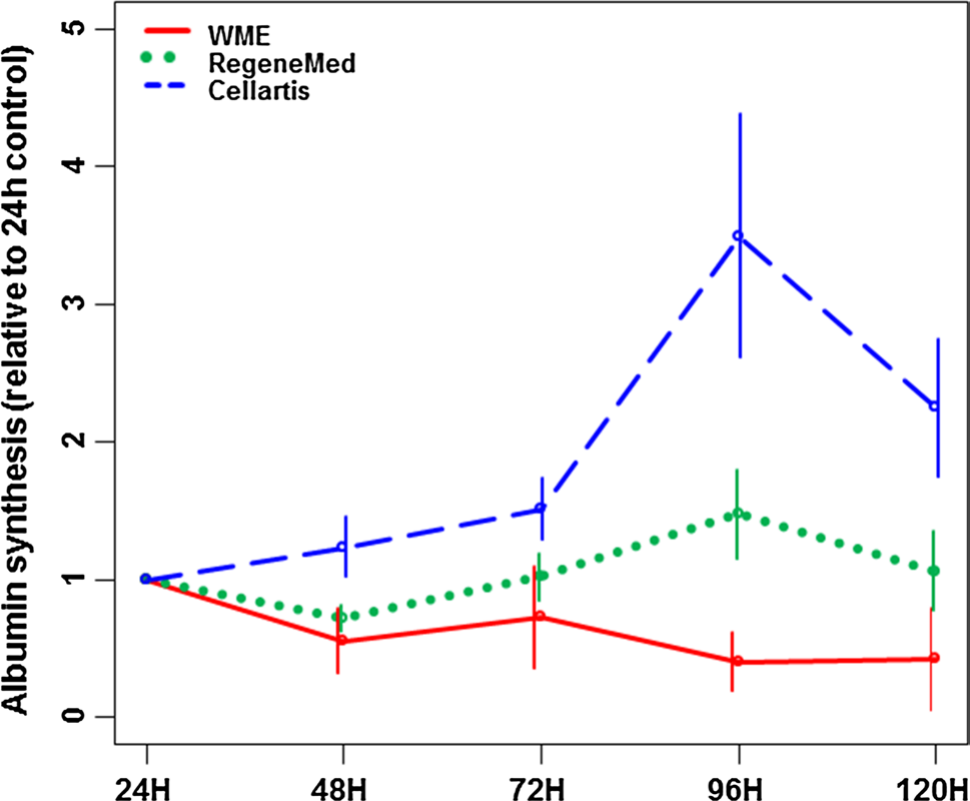
Albumin synthesis over time by hPCLS incubated in WME (*red line*), RegeneMed^®^ (*green dotted line*) or Cellartis^®^ (*blue dashed line*). Date are expressed as relative value to 24-h control (±SEM) (color figure online)

Incubation time (*p* = 0.02) and composition of medium (*p* < 0.001) had a significant effect on albumin synthesis in hPCLS. The effect of incubation time was different across media (*p* for interaction: 0.007), with a significant increase in albumin synthesis over time in Cellartis^®^ and a constant level of synthesis in RegeneMed^®^ and WME (Fig. 6).

### Gene expression of metabolic and transporter-related genes

To further identify potential changes in drug metabolism, transport, and toxicology-related pathways during culture, we performed a transcriptomic analysis of slices incubated for 120 h in Cellartis^®^ medium. As the functional results showed the best maintenance of hPCLS functionality in Cellartis^®^ medium but a significant decline in WME and RegeneMed^®^, we did not perform transcriptomic analysis of hPCLS incubated in the latter two media. Following 120 h of incubation in Cellartis^®^ medium, a limited proportion, 704 genes were shown to be up- or downregulated (*p* < 0.05) compared to the 0 h control slices, out of which 57.5 % were upregulated. In order to investigate the stability of hPCLS during incubation with respect to expression of genes related to xenobiotic metabolism and drug transport, we listed the significantly regulated genes involved in phase I and II metabolism (Table 1) as well as in drug transport. Moreover, pathway analysis showed that the majority of pathways related to liver damage such as cholestasis, steatosis, apoptosis, necrosis, or mitochondria damage were not up- or downregulated in hPCLS after 5 days of incubation in Cellartis^®^ medium. Some pathways, however, were shown to be differentially regulated, among which oxidative stress and fibrosis. Therefore, we listed the differentially expressed genes involved in oxidative stress and fibrosis development in Tables 2 and 3. The list of top 20 significantly regulated pathways is given in supplementary Fig. 7.

**Table 1:** Significantly regulated genes involved in phase I and phase II metabolism and metabolism signaling

Gene title	Gene symbol	Fold change	*P* value
Alcohol dehydrogenase 5 (Class III)	ADH5	1.6	0.037
Aryl hydrocarbon receptor	AHR	−1.7	0.046
Aldehyde dehydrogenase 1 family, member A1	ALDH1A1	4.0	0.009
Aldehyde dehydrogenase 3 family, member A2	ALDH3A2	3.5	0.008
Aldehyde dehydrogenase 8 family, member A1	ALDH8A1	2.2	0.047
Calcium/calmodulin-dependent protein kinase II beta	CAMK2B	−3.3	0.046
Carboxylesterase 2	CES2	2.4	0.043
Cbp/P300-interacting transactivator, with Glu/Asp-Rich carboxy-terminal domain, 2	CITED2	4.1	0.029
Cytochrome P450, family 1, subfamily A, polypeptide 1	CYP1A1	136.6	0.0002
Cytochrome P450, family 1, subfamily A, polypeptide 2	CYP1A2	11.1	0.019
Cytochrome P450, family 1, subfamily B, polypeptide 1	CYP1B1	14.1	0.006
Cytochrome P450, family 24, subfamily a, polypeptide 1	CYP24A1	30.2	0.0005
Cytochrome P450, family 26, subfamily a, polypeptide 1	CYP26A1	4.7	0.017
Cytochrome P450, family 26, subfamily b, polypeptide 1	CYP26B1	2.9	0.033
Cytochrome P450, family 2, subfamily a, polypeptide 6	CYP2A6	10.3	0.041
Cytochrome P450, family 2, subfamily b, polypeptide 6	CYP2B6	2.9	0.037
Cytochrome P450, family 2, subfamily c, polypeptide 18	CYP2C18	4.0	0.017
Cytochrome P450, family 2, subfamily c, polypeptide 19	CYP2C19	6.0	0.046
Cytochrome P450, family 2, subfamily c, polypeptide 8	CYP2C8	2.6	0.041
Cytochrome P450, family 2, subfamily c, polypeptide 9	CYP2C9	4.0	0.043
Cytochrome P450, family 3, subfamily a, polypeptide 4	CYP3A4	11.7	0.047
Eukaryotic translation initiation factor 2-alpha kinase 3	EIF2AK3	−1.8	0.047
Fas cell surface death receptor	FAS	2.8	0.021
Growth arrest and DNA-damage-inducible, beta	GADD45B	−11.0	0.011
Glutathione S-transferase alpha 1	GSTA1	20.4	0.044
Glutathione S-transferase alpha 2	GSTA2	30.9	0.019
Glutathione S-transferase alpha 3	GSTA3	1.8	0.035
Glutathione S-transferase alpha 5	GSTA5	8.9	0.047
Glutathione S-transferase Mu 4	GSTM4	2.0	0.029
Glutathione S-transferase Omega 1	GSTO1	3.6	0.017
Microsomal glutathione S-transferase 1	MGST1	4.1	0.014
Microsomal glutathione S-transferase 3	MGST3	1.8	0.034
N-Acetyltransferase 8	NAT8	8.2	0.008
Nuclear receptor co-activator 7	NCOA7	−2.0	0.039
N-Deacetylase/N-Sulfotransferase (heparan glucosaminyl) 2	NDST2	−1.9	0.035
Nuclear factor I/X	NFIX	−2.3	0.047
NAD(P)H dehydrogenase, quinone 1	NQO1	10.4	0.0006
Phosphoenolpyruvate carboxykinase 2	PCK2	4.3	0.024
Peroxisome proliferator-activated receptor gamma, co-activator 1 alpha	PPARGC1A	−3.7	0.021
Protein phosphatase 2, regulatory subunit a, beta	PPP2R1B	−5.3	0.027
Retinoic acid receptor, alpha	RARA	−2.7	0.030
Related RAS viral (R-Ras) oncogene homolog 2	RRAS2	2.2	0.039
Retinoid X receptor, gamma	RXRG	−2.3	0.049
Sp1 transcription factor	SP1	−1.9	0.047
SRC proto-oncogene, non-receptor tyrosine kinase	SRC	2.8	0.024
Ubiquitin carboxyl-terminal esterase L1 (ubiquitin thiolesterase)	UCHL1	4.6	0.01
UDP glucuronosyltransferase 1 family, polypeptide A1	UGT1A1	17.9	0.008
UDP glucuronosyltransferase 1 family, polypeptide A3	UGT1A3	3.0	0.037
UDP glucuronosyltransferase 1 family, polypeptide A4	UGT1A4	7.8	0.021
UDP glucuronosyltransferase 1 family, polypeptide A6	UGT1A6	10.2	0.024
UDP glucuronosyltransferase 2 family, polypeptide A3	UGT2A3	6.4	0.01
UDP glucuronosyltransferase 2 family, polypeptide B11	UGT2B11	4.4	0.011
UDP glucuronosyltransferase 2 family, polypeptide B15	UGT2B15	11.0	0.011
UDP glucuronosyltransferase 2 family, polypeptide B17	UGT2B17	7.3	0.012
UDP glucuronosyltransferase 2 family, polypeptide B4	UGT2B4	4.1	0.019
UDP glucuronosyltransferase 2 family, polypeptide B7	UGT2B7	2.2	0.033

**Table 2:** Significantly regulated genes involved in oxidative stress response

Gene title	Gene symbol	Fold change	*P* value
Actin, beta	ACTB	2.5	0.029
Actin gamma 1	ACTG1	2.2	0.017
Aldo–Keto reductase family 7, member A3 (aflatoxin aldehyde reductase)	AKR7A3	4.0	0.039
Activating transcription factor 4	ATF4	−2.7	0.018
Carbonyl reductase 1	CBR1	2.1	0.023
Chemokine (C–C Motif) ligand 5	CCL5	−2.0	0.044
DnaJ (Hsp40) homolog, subfamily B, member 11	DNAJB11	−3.4	0.012
DnaJ (Hsp40) homolog, subfamily C, member 12	DNAJC12	−3.8	0.027
DnaJ (Hsp40) homolog, subfamily C, member 3	DNAJC3	−2.0	0.043
Ferritin, heavy polypeptide 1	FTH1	3.0	0.046
Glutathione synthetase	GSS	1.9	0.041
3-Hydroxyacyl-CoA dehydratase 3	HACD3	2.6	0.009
Interleukin 10	IL10	−1.8	0.042
Peroxiredoxin 2	PRDX2	1.7	0.042
Peroxiredoxin 3	PRDX3	2.7	0.024
Signal transducer and activator of transcription 3 (acute-phase response factor)	STAT3	−2.0	0.036
Thioredoxin	TXN	2.6	0.043

**Table 3:** Significantly regulated genes involved in fibrosis development

Gene title	Gene symbol	Fold change	*P* value
BMP and activin membrane-bound inhibitor	BAMBI	2.0	0.037
Collagen, type XVI, alpha 1	COL16A1	2.1	0.041
Collagen, type I, alpha 1	COL1A1	12.3	0.006
Collagen, type I, alpha 2	COL1A2	9.1	0.015
Collagen, type III, alpha 1	COL3A1	8.7	0.021
Collagen, type VI, alpha 3	COL6A3	7.2	0.006
Decorin	DCN	2.3	0.038
Fibronectin 1	FN1	1.9	0.025
Interferon (alpha, beta, and omega) receptor 1	IFNAR1	−2.2	0.041
Insulin-like growth factor 1 (somatomedin C)	IGF1	−3.4	0.039
Insulin-like growth factor 2	IGF2	−3.2	0.017
Insulin-like growth factor binding protein 6	IGFBP6	1.8	0.026
Interleukin 4 receptor	IL4R	−3.8	0.019
Lipopolysaccharide binding protein	LBP	−1.7	0.037
Leptin	LEP	−1.9	0.039
Lectin, galactoside-binding, soluble, 3	LGALS3	3.5	0.017
Lumican	LUM	6.4	0.022
SMAD family member 4	SMAD4	1.6	0.048
Signal transducer and activator of transcription 1, 91 kDa	STAT1	−2.0	0.041
Synovial apoptosis inhibitor 1, synoviolin	SYVN1	−1.9	0.026
Transforming growth factor, alpha	TGFA	2.2	0.021
Transforming growth factor, beta receptor II	TGFBR2	2.4	0.035
Vitronectin	VTN	−1.7	0.047

### Phase I and II metabolism

Table 1 shows the genes involved in drug metabolism and its regulation that were significantly regulated after 5 days of incubation. The gene expression of many of the phase I metabolism enzymes was stable in hPCLS during 5 days of incubation. Remarkably, CYPs known to play an important role in drug metabolism, such as CYP1A1, CYP1A2, CYP3A4, CYP2B6, CYP2C9, CYP2C19, and CYP2C8 were upregulated during incubation. Monooxygenases (FMO) or glutathione peroxidase was not affected after 5 days of incubation. Among the aldehyde dehydrogenases, ALDH1A1, ALDH3A2, and ALDH8A1 were upregulated after 5 days, and among the alcohol dehydrogenases only ADH5 was upregulated, while all other ALDH’s and ADH’s were unchanged. Some of the genes coding for phase II metabolism enzymes were upregulated after 5 days of incubation, such as gluthatione S-transferases (GST’s) and UGT’s. SULTs, methyltransferases (MTs), and N-acetyltransferases (NATs, with the exception of NAT8) were not regulated. Most of the transcription factors involved in the regulation of drug metabolizing enzymes, such as PXR, CAR, GR, and FXR were unchanged, only AhR was somewhat downregulated (1.7 fold).

### Transporters

Drug uptake (SLC’s) and excretion (MDR’s and MRP’s) transporters are important determinants for the intracellular exposure to drugs and their metabolites. The expression of the genes coding for the main human drug transporters (MRP’s and SLC’s) were unchanged after 5 days of incubation, with the exception of MRP5, which was slightly (1.7-fold) upregulated. The changes in expression of other transporters, not directly involved in drug transport, were limited. For example, the expression of ABCA1, responsible for the efflux of cholesterol, and SLC27A5, responsible for fatty acid transport, were moderately (twofold to fourfold) downregulated, whereas the expression of ATP2C1, responsible for calcium transport, was moderately (2.7 fold) upregulated (Table 5, Supplementary materials).

### Oxidative stress

During incubation, a limited number of genes involved in oxidative stress response was regulated (Table 2). The twofold to fourfold upregulation of aldo–keto reductase AKR7A3 (involved in the detoxification of aldehydes and ketones), carbonyl reductase CBR1 (involved in the detoxification of carbonyl compounds, such as quinones, prostaglandins, and various xenobiotics), glutathione synthetase GSS (involved in glutathione synthesis, an important antioxidant), peroxiredoxin PRDX2 and PRDX3 (antioxidant enzymes which reduce hydrogen peroxide and alkyl hydroperoxides), and thioredoxin TXN (involved in many redox reactions) indicates that slices undergo some moderate oxidative stress and respond by upregulating defense mechanisms. However, some stress markers, such as CCL5, IL10, and STAT3 were downregulated after 5 days of incubation.

### Fibrosis

Pathway analysis showed that some of the genes involved in fibrosis development were regulated after 5 days of incubation. For example, collagen genes COL16A1, COL1A1, COL3A1, COL6A3, FN1, decorin, and lumican were shown to be upregulated after 5 days (Table 3). COL’s and FN1 are responsible for collagen and fibronectin synthesis, respectively, while decorin and lumican play a role in collagen fibril assembly. Moreover, several genes involved in TGF signaling pathways, such as BAMBI, SMAD4, TGFA, and TGFBR2, were moderately upregulated. These findings are in line with the morphological observation of an increase in collagen deposition in slices after 5 days of incubation.

## Discussion

PCLS have been extensively used for drug toxicity studies and are considered to most closely represent the original liver, retaining all the liver cells in their natural environment. Moreover, the use of hPCLS makes it possible to avoid extrapolation steps from animal to human studies, since it is recognized that results obtained from animal-based models cannot be directly extrapolated to humans, due to among others the differences in metabolism and transport of xenobiotics (Chu et al. 2013; Karthikeyan et al. 2016).

The viability of hPCLS was preserved during 5 days of incubation in Cellartis^®^ and RegeneMed^®^ medium, but not in WME, which was different compared to our previous studies on rat PCLS, where slices incubated in WME retained their viability during prolonged incubation similar to slices incubated in RegeneMed^®^. hPCLS incubated in WME decreased in protein content following incubation, likely due to the decline in their viability and cell death. hPCLS incubated in RegeneMed^®^, however, maintained their protein content during incubation, whereas the protein content in slices incubated in Cellartis^®^ medium gradually increased somewhat during incubation, which might indicate protein synthesis and/or cell proliferation. Cell proliferation can also be responsible for the observed ca. 20–40 % increase in thickness of the slices during incubation in RegeneMed^®^ and Cellartis^®^ medium, which was far less than previously observed in rat PCLS (Starokozhko et al. 2015). Even though the slices increased in thickness during incubation, the oxygen penetration to the inner cell layers was sufficient, since no necrotic/hypoxic bands of cells were seen in the inner part of the slices. Only occasional necrotic areas were observed in hPCLS cultured in Cellartis^®^ and RegeneMed^®^ medium, whereas slices incubated in WME had large necrotic regions with pycnotic nuclei. The formation of a new cell layer around the slices during culture has been already described before for rat PCLS by us (Starokozhko et al. 2015). This newly formed cells layer in hPCLS was positive for vimentin indicating the mesenchymal origin of these cells.

The hPCLS incubated in RegeneMed^®^ and Cellartis^®^ medium showed good maintenance of glucose homeostasis and albumin synthesis, whereas the slices in WME partially lost these capacities, which can at least partly be explained by the absence of insulin in WME, whereas both the other media contain insulin.

Biotransformation in the liver can lead to detoxification or toxification of a drug and liver transporters can increase or reduce the actual intracellular exposure to a xenobiotic. Therefore, the expression and functionality of metabolic enzymes and transporters in the human in vitro model at the levels comparable to in vivo values is an important requirement for toxicity studies. The stability of expression of genes involved in drug metabolism and transport, as well as stress and toxicity responses have been characterized up to 24 h in hPCLS culture before (Elferink et al. 2011). However, the stability of these genes and, importantly, the activity of phase I and II metabolic enzymes during prolonged hPCLS culture has never been fully investigated. This is an particularly important requirement for toxicity studies that require prolonged exposure to the drug. Therefore, we characterized the changes in phase I and II metabolic enzymes both on gene expression and functional levels. Moreover, we assessed the changes in hPCLS viability, morphology and functionality following 5 days of incubation in three different media.

Here, for the first time, the stability of the activity of liver enzymes involved in drug metabolism was achieved during prolonged 5 days incubation in hPCLS. Earlier studies showed a progressive decrease in CYP apoprotein levels and activity levels during 72-h incubation (Renwick et al. 2000). In our study the activity of the tested CYP isoforms was stable in Cellartis^®^ Hepatocyte Maintenance medium, with a slight decrease in CYP2C9 as the only exception. Glucuronidation and sulfation rates also remained stable in hPCLS incubated in Cellartis^®^ Hepatocyte Maintenance medium during 5 days. The activity of various phase I and phase II metabolic enzymes in hPCLS cultured in WME or RegeneMed^®^ medium, however, declined in time. Metabolism of 7EC increased over time in slices incubated in Cellartis^®^ Hepatocyte Maintenance medium, which is in line with the upregulation of CYP1A2, one of the enzymes responsible for 7-EC oxidation (Yamazaki et al. 1996). The significant upregulation of CYP1A activity has to be taken into account during toxicity studies which involve this isoenzyme, since it might lead to over- or underestimation of toxicity of a tested drug depending whether its oxidation by CYP1A leads to toxification or detoxification of a parent compound, respectively.

Transcriptomics analysis of hPCSL incubated in Cellartis^®^ medium showed that transcriptional effects were only observed in a smaller fraction of the global transcriptome (704 genes out of 31,000), and the changes in gene expression of phase I and II metabolic enzymes and drug transporters were limited. Among the CYPs, 13 isoforms were upregulated and none of the CYPs were downregulated in hPCLS after 5 days of incubation. This is a major achievement as previously downregulation of CYP expression was reported during prolonged incubation (Vickers et al. 2011). As the expression of PXR and AhR is not changed, the significant upregulation of the PXR and AhR signaling pathways, which is based on the upregulation of the CYP enzymes, might be due to either the presence or increased activity of co-regulators. The slight decline in CYP2C9 activity is not in line with the fourfold increase in CYP2C9 gene expression. A decreased activity of the NADPH-cytochrome P450 reductase is unlikely to be the cause of this discrepancy, as the other Cytochrome P450 isoenzymes show constant or even increased activity. The expression of phase II metabolic enzymes was upregulated (UGTs and GSTs) or unchanged (SULTs, NATs, MTs) after 5 days. None of the phase II metabolism enzymes were downregulated during incubation. Also the increased expression of the UGTs did not result in increased enzyme activity. It cannot be excluded that the synthesis of the co-substrate is a rate-limiting factor for conjugation. Moreover, the expression of all the main drug transporters remained constant during 5 days of incubation, indicating that the exposure of the cells to the drugs and metabolites is representative for the in vivo situation.

Most of the pathways known to be involved in liver toxicity were unchanged in hPCLS during 5 days of incubation, with the exception of liver fibrosis and oxidative stress. Oxidative stress in PCLS is a known response to the slicing procedure and culture conditions, in particular, the high oxygen tension (Martin et al. 2002). In our study, the regulation of genes involved in oxidative stress pathways was slight or moderate (fold induction <4). On the other hand, upregulation of antioxidant and other detoxification pathways indicates that the natural defense mechanisms can be activated in hPCLS during prolonged incubation. The development of fibrosis in hPCLS during incubation was reflected both in collagen deposition, as well as in upregulation of genes involved in fibrogenic pathways, such as COLs and FN1. These findings are in line with our previous studies on liver slices incubated in WME, which described the suitability of hPCLS to study the effects and toxicity of antifibrotic drugs (van de Bovenkamp et al. 2008; Westra et al. 2014).

Our findings that Cellartis^®^ Hepatocyte Maintenance medium maintains high metabolic functionality and viability of hPCLS for 5 days suggests that this medium prevents the de-differentiation which occurs in hPCLS in the commonly used culture medium like WME, which is characterized by a rapid loss of functionality, possibly by lack of specific differentiation signaling molecules. Interestingly, Cellartis^®^ Hepatocyte Maintenance medium was initially developed for culturing hepatocytes derived from human pluripotent stem cells. In stem-cell-derived hepatocytes, it promotes a mature hepatocyte phenotype, e.g., expression of adult drug metabolizing enzymes such as CYP2C9 and CYP3A4 in stem-cell-derived hepatocytes from day 21 after start of differentiation and onwards (Ghosheh et al. 2016), without the presence of specific PXR or CAR inducers. Further studies are currently performed to test whether hPCLS can be maintained for longer than 5 days in Cellartis^®^ Hepatocyte Maintenance medium which would open up for long-term use of hPCLS. In addition, it would be interesting to attempt to adjust the medium composition in a way that leads to a somewhat lower CYP1A activity and thus a more balanced CYP activity profile.

In conclusion, we showed that hPCLS retain their viability and functionality during 5 days of incubation. The type of incubation medium influences liver viability, morphology, and functions, with the best results shown with Cellartis^®^ Hepatocyte Maintenance medium. Synthesis functions, activity and gene expression of phase I and II metabolic enzymes did not decline during 120-h incubation in Cellartis^®^ medium, with the CYP2C9 activity as the only exception. Moreover, gene expression changes in hPCLS during incubation were limited and mostly related to the cytoskeleton remodeling, fibrosis and moderate oxidative stress, whereas other pathways involved in liver toxicity were not regulated. The expression of genes involved in drug transport was also unchanged during 5 days, which is an important factor that determines the final intracellular xenobiotic exposure. Taken together, we conclude that hPCLS are a valuable human in vitro model for toxicological and pharmacological studies and can be used for studies that require prolonged xenobiotic exposure. Moreover, the use of human slices enables direct identification of toxicological effects of drugs relevant for human, thereby reducing experimental animal use and facilitating animal to human extrapolation steps.

## Electronic supplementary material

Below is the link to the electronic supplementary material.

Supplementary material 1 (DOCX 231 kb)
