# Supplementary material for: Maintenance of drug metabolism and transport functions in human precision-cut liver slices during prolonged incubation for 5 days
Source: Arch Toxicol. 2016 Oct 7;91(5):2079–92. doi: 10.1007/s00204-016-1865-x (PMC5399048; doi:10.1007/s00204-016-1865-x)
Supplement: Supplementary file 1 — Supplementary material 1 (DOCX 231 kb) [file 204_2016_1865_MOESM1_ESM.docx]

***Table 4.*** ***Human liver donor characteristics***

| Number | Type of liver | Gender | Age |
| --- | --- | --- | --- |
| 1 | Reduced size liver transplantation | female | 63 |
| 2 | Hepatectomy for the removal of carcinoma | male | 64 |
| 3 | Reduced size liver transplantation | female | 20 |
| 4 | Donated after cardiac death | male | 71 |
| 5 | Reduced size liver transplantation | male | 54 |

***Figure 7.*** ***Top 20 significantly regulated pathways in hPCLS following 5 days of incubation***


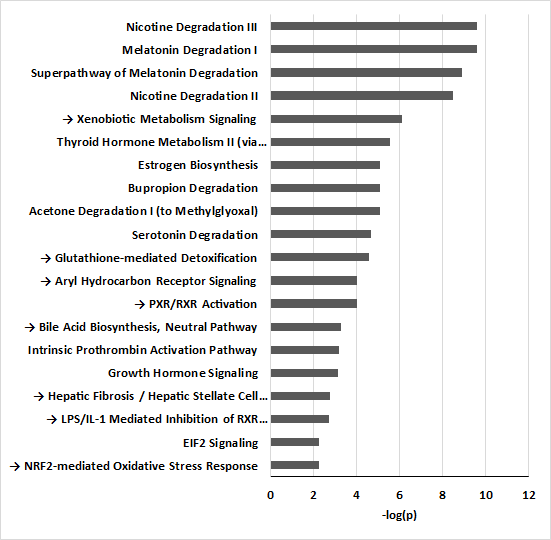


***Table 5. Significantly regulated genes transporters***

| Gene title | Gene symbol | Protein name | Fold change | P value |
| --- | --- | --- | --- | --- |
| ATP-Binding Cassette, Sub-Family A (ABC1), Member 1 | ABCA1 |  | -2.0 | 0.019 |
| ATP-Binding Cassette, Sub-Family B (MDR/TAP), Member 10 | ABCB10 |  | 2.2 | 0.023 |
| ATP-Binding Cassette, Sub-Family C (CFTR/MRP), Member 5 | ABCC5 | MRP5 | 1.7 | 0.049 |
| ATPase, Ca++ Transporting, Type 2C, Member 1 | ATP2C1 |  | 2.7 | 0.019 |
| ATPase, H+ Transporting, Lysosomal 9kDa, V0 Subunit E1 | ATP6V0E1 |  | 1.7 | 0.049 |
| Solute Carrier Family 1 (Glial High Affinity Glutamate Transporter), Member 2 | SLC1A2 | GLT1-EAAT2 | -3.1 | 0.0099 |
| Solute Carrier Family 27 (Fatty Acid Transporter), Member 5 | SLC27A5 | FATP5 | -3.2 | 0.036 |
